# Global tropospheric ozone responses to reduced NO_*x*_ emissions linked to the COVID-19 worldwide lockdowns

**DOI:** 10.1126/sciadv.abf7460

**Published:** 2021-06-09

**Authors:** Kazuyuki Miyazaki, Kevin Bowman, Takashi Sekiya, Masayuki Takigawa, Jessica L. Neu, Kengo Sudo, Greg Osterman, Henk Eskes

**Affiliations:** 1 Jet Propulsion Laboratory, California Institute of Technology, Pasadena, CA, USA.; 2 Joint Institute for Regional Earth System Science and Engineering, University of California, Los Angeles, 4242 Young Hall, 607 Charles E. Young Drive East, Los Angeles, CA 90095-7228, USA.; 3 Japan Agency for Marine-Earth Science and Technology, Yokohama 236-0001, Japan.; 4 Graduate School of Environmental Studies, Nagoya University, Nagoya, Japan.; 5 Royal Netherlands Meteorological Institute, De Bilt, Netherlands.

## Abstract

Efforts to stem the transmission of coronavirus disease 2019 (COVID-19) led to rapid, global ancillary reductions in air pollutant emissions. Here, we quantify the impact on tropospheric ozone using a multiconstituent chemical data assimilation system. Anthropogenic NO_*x*_ emissions dropped by at least 15% globally and 18 to 25% regionally in April and May 2020, which decreased free tropospheric ozone by up to 5 parts per billion, consistent with independent satellite observations. The global total tropospheric ozone burden declined by 6TgO_3_ (∼2%) in May and June 2020, largely due to emission reductions in Asia and the Americas that were amplified by regionally high ozone production efficiencies (up to 4 TgO_3_/TgN). Our results show that COVID-19 mitigation left a global atmospheric imprint that altered atmospheric oxidative capacity and climate radiative forcing, providing a test of the efficacy of NO_*x*_ emissions controls for co-benefiting air quality and climate.

## Teaser

Global ozone plunged during the COVID-19 pandemic: a glimpse into future benefits for air quality and climate.

## INTRODUCTION

To slow the transmission of coronavirus disease 2019 (COVID-19), numerous countries worldwide have imposed lockdown measures that severely limit personal mobility, leading to reductions in overall economic activity (*1*). These restrictions on human activity were designed to alleviate the strain on the health care system from COVID-19 (*2*) but also had the ancillary impact of rapid air pollutant emission reductions. Changes in greenhouse gas and pollutant emissions have been estimated using activity data such as mobility metrics (*3*–*5*), with global NO_*x*_ emissions estimated to have declined as much as 30% in April (*4*). However, these estimates are highly uncertain, as activity data are incomplete, and substantial assumptions are needed to relate these data to the partitioning and magnitude of emissions.

Substantial impacts on regional and global air quality during the COVID-19 period have also been demonstrated using various in situ and satellite measurements (*6*–*10*). A study using the spatially limited set of global surface in situ air quality measurement networks estimated declines in population-weighted concentration of 60% for surface nitrogen dioxide (NO_2_) and 31% for particulate matter smaller than 2.5 μm (PM2.5), and marginally notable increases of 4% in ozone between the beginning of the lockdowns and May 15 (*11*). These estimates highlight the different responses of surface concentrations for different species and the strong regional dependence of the response, but because of the sparseness of the in situ network, they do not provide a truly global picture of the pandemic’s impact on atmospheric composition.

Satellite measurements such as those from the TROPOspheric Monitoring Instrument (TROPOMI) have captured the rapid reductions in tropospheric NO_2_ columns, as well as in other species, associated with global COVID-19 lockdown measures (*7*, *12*). However, the inference of emissions from the observed concentrations must account for variations in atmospheric transport, chemical environment, and meteorology (*13*, *14*). Furthermore, the response of ozone and PM2.5 to reduced NO_*x*_ emissions is of particular interest because of their effects on human health (*15*), and in the case of ozone, its crucial role in tropospheric chemistry and chemistry-climate interactions as the third most important anthropogenic greenhouse gas in the atmosphere (*16*, *17*). Tropospheric ozone is produced from its precursors, primarily NO_*x*_ and volatile organic compounds (VOCs), through nonlinear chemical processes. Because of the dependency of ozone production on photochemical environment, its response to emission reductions is expected to vary substantially on the basis of timing and location. However, the current in situ observing network is too sparse to capture this variable response. Furthermore, the tendency toward sampling highly populated areas could lead to biased estimations when extrapolating from regional to global scales because of local titration effects. Although satellite measurements provide much denser sampling than surface networks, the lack of consistent long-term records of ozone from satellites (*18*) and natural variability in pollutants such as those from biomass burning make it difficult to detect COVID-19 signals in observed ozone concentrations.

In the decade before the COVID-19 pandemic, many countries implemented environmental policies to reduce human health risks associated with poor air quality. These policies largely focused on regulating air pollutant emissions through changes in human activity and through increased efficiency (i.e., technology). However, the actual response of atmospheric composition to these policies cannot be directly measured because factors other than changes in emissions, such as climatic conditions, meteorology, and the background chemical state, affect air pollutant levels and can exhibit long-term variations that confound detection of emission-driven changes (*13*, *19*). The COVID-19 period, however, is unique in terms of the speed and magnitude of emission changes and the fact that they occurred over a very short period of time, thus limiting the need to disentangle the effects of emissions from long-term variability and ensuring that the response could be measured via a stable, consistent observational network. COVID-19 therefore represents a “scenario-of-opportunity” that informs our understanding of how atmospheric composition responds to rapid and large reductions in human activity and concomitant air pollutant emissions. Analysis of the atmospheric composition response to COVID-19 lockdown measures thus provides important information on effective environmental policy-making aimed at improving air quality. Most relevant for this study, tropospheric ozone and aerosols also affect radiative forcing; therefore, their response to changing emissions also sheds light on air quality–climate cobenefits (*16*).

This study quantifies the response of global tropospheric ozone to the unprecedented NO_*x*_ emission reductions associated with COVID-19. This analysis is made possible by a state-of-the-art multiconstituent satellite data assimilation system (*20*) that ingests multiple satellite observations to simultaneously optimize concentrations and emissions of various trace gas species, while taking their complex chemical interactions into account. This framework was already used to quantify the surface air quality response to Chinese COVID-19 lockdown measures (*21*).

## RESULTS

### Global NO_*x*_ emission reductions

**Fig. 1:**
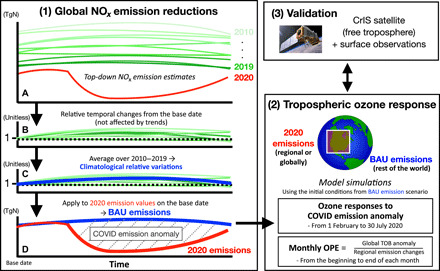
Schematic diagram of the methodology used in this study. (1) (**A**) The top-down 2010–2019 emissions obtained from the chemical data assimilation (green lines) were used to (**B**) evaluate relative temporal emission changes from the base date (February 1 and January 10 for China only) through July 31 each year. (**C**) The calculated relative temporal emission changes were averaged over the 10 years (2010–2019) to obtain climatological relative emission variations (solid blue line). (**D**) The climatological variations were applied to the 2020 emission (solid red line) values on the base date to obtain the BAU emissions for 2020 (solid blue line) and then compared with the 2020 emissions to estimate the COVID emission anomaly. (2) The COVID-19 ozone response through February to July 2020 and monthly ozone production efficiency (OPE) estimated from the beginning to end of each month were estimated from model simulations by replacing the BAU emissions with the 2020 emissions for each region or globally. TOB, tropospheric ozone burden. (3) The evaluated ozone response was compared with the observed changes from the Cross-Track Infrared Sounder (CrIS) satellite and surface observations.

Anthropogenic NO_*x*_ emission reductions linked to the COVID-19 pandemic were estimated as the difference between baseline “business as usual” (BAU) emissions, obtained by aggregating 2010–2019 emissions from our decadal chemical reanalysis constrained by multiple satellite measurements (*20*), and 2020 emissions derived from the same system, using 2020 TROPOMI NO_2_ observations. The BAU emissions were adjusted to 2020 values using the difference between the 2010–2019 baseline and 2020 emissions on February 1, when economic activity was not yet substantially affected by COVID-19 mitigation for most countries. For China, however, where the first government-imposed lockdown occurred earlier than in the rest of the world, the difference in emissions on January 10 is used to obtain the BAU emissions. Therefore, the 2020 COVID-19 emission anomaly, estimated as difference between the BAU and COVID-19 emissions, does not include the influence of climatological seasonal changes in anthropogenic emissions, such as use of winter heating, nor does it include interannual changes from years before 2020 (see Materials and Methods and Fig. 1 for further information). Biomass burning and soil NO_*x*_ emissions, as well as areas that were heavily affected by clouds and at high latitudes (higher than 55°), were removed from the data assimilation analysis. The a priori emissions used in the data assimilation system have limited representation of actual ship tracks, which hinders evaluation of ship emission changes; NO_*x*_ emissions over oceans were thus removed as well. Although our analysis covers about 75% of the global total NO_*x*_ emissions, actual emission changes at country or global scales are likely larger than our estimates because of the unrepresented areas. Uncertainties on the COVID-19 emission anomalies were estimated from the interannual variability in the BAU emissions, model errors, and short-term variability of the emissions (see Materials and Methods for further information).

The NO_2_ in the model simulation using the optimized emissions exhibits consistent variations with the assimilated satellite NO_2_ column measurements globally (fig. S1) and surface NO_2_ measurements over North America, Europe, the Middle East, and East Asia (table S1). The remaining model negative NO_2_ bias against the surface measurements is likely a consequence of the reported negative bias in the assimilated TROPOMI NO_2_ measurements (*22*). Meanwhile, the regional or country mean tropospheric NO_2_ columns show notably different seasonal and spatial changes than the total NO_*x*_ emissions (fig. S1) due to varying influences of nonlinear chemical and meteorological conditions. For example, tropospheric NO_2_ concentrations naturally decrease from winter through summer as a result of photochemical processes, even without any reduction in emissions.

**Fig. 2:**
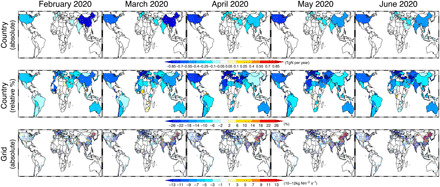
Spatial distributions of the monthly mean NO_*x*_ emission reductions due to the COVID-19 lockdowns. The COVID NO_*x*_ emission anomaly in February to June 2020 was estimated from differences between the 2020 and BAU emissions. Results are shown for the absolute changes in country total emissions (in TgN per year, top), relative changes in country total emissions (in %, middle), and absolute changes in grid-scale emissions (in 10^−12^kg Nm^−2^ s^−1^, bottom). The model grid points that were not analyzed because of unstable emission estimates and fire influences are shown in gray. N America, North America; S America, South America; ME + W Asia, Middle East and West Asia.

**Fig. 3:**
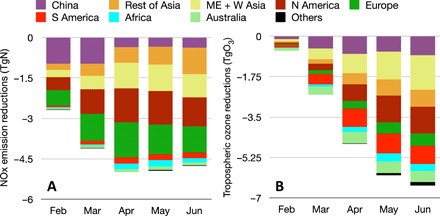
Reductions in anthropogenic NO_*x*_ emissions and global TOB. Monthly mean global and regional total changes in (**A**) NO_*x*_ emissions (in TgN per year) due to the COVID-19 lockdowns and in (**B**) global TOB (in TgO_3_) due to regional NO_*x*_ emission changes are shown for Africa, Europe, Australia, the Middle East and West Asia, the rest of Asia, South America, North America, China, and other regions.

We estimate that global total anthropogenic NO_*x*_ emissions in 2020 were reduced by 9.0 ±1.5% relative to the global total anthropogenic emissions (12.8 ±2.1% relative to the analyzed areas total anthropogenic emissions) in February, 12.7± 1.5% (17.8 ± 2.1%) in March, 14.8± 2.3% (21.2 ±3.3%) in April, 15.0 ±1.8% (21.8 ±2.6%) in May, and 13.9±1.8% (20.8 ± 2.6%) in June relative to the BAU emissions (Table 1 and Figs. 2 and 3A). In February, the reduction in emissions from China made the largest contribution (36%) to the global NO_*x*_ anomaly, whereas the contributions from other regions are larger from March to June, when China relaxed its restrictions. Regional total anthropogenic emissions dropped by 18 to 25% in April and May across Europe, North America, and the Middle East and West Asia. Africa and South America also show clear but moderate reductions in emissions (∼5 to 10%) in April and May, with substantial spatial variations within the regions. The peak reduction in global total NO_*x*_ emissions of about 5 TgN/year is almost the same as the climatological annual anthropogenic emissions from Europe in our estimates. In many regions, the early emission reductions in February and March suggest that activity likely started decreasing even before actual implementation of lockdown measures, as further discussed below.

**Table 1:** Monthly mean values of global and regional total surface NO_*x*_ emission changes (in %) due to the COVID-19 restrictions. The 1-sigma uncertainties, estimated from the SD of the multiyear BAU emissions, are also shown. W Asia, West Asia; S America, South America; N America, North America.

**Region**	**February**	**March**	**April**	**May**	**June**
Globe	−9.0 ± 1.5	−12.7 ± 1.5	−14.8 ± 2.3	−15.0 ± 1.8	−13.9 ± 1.8
Africa	−1.8 ± 3.7	−2.1 ± 4.2	−9.9 ± 4.4	−10.3 ± 4.0	−6.7 ± 4.1
Europe	−10.3 ± 4.1	−16.5 ± 4.6	−19.3 ± 5.8	−18.7 ± 5.6	−13.8 ± 3.6
Australia	−10.2 ± 4.0	−12.8 ± 5.3	−14.6 ± 5.7	−15.7 ± 6.2	−15.9 ± 7.4
Middle East + W Asia	−8.3 ± 4.8	−14.8 ± 6.8	−24.1 ± 9.7	−24.8 ± 9.6	−21.7 ± 10.6
Rest of Asia	−4.0 ± 1.3	−7.4 ± 1.6	−9.4 ± 2.6	−10.6 ± 2.1	−14.4 ± 2.1
S America	−3.3 ± 1.5	−7.0 ± 1.8	−10.2 ± 2.5	−10.2 ± 2.4	−10.3 ± 2.9
N America	−9.6 ± 2.6	−16.1 ± 4.3	−20.7 ± 6.2	−20.1 ± 5.5	−17.5 ± 4.6
China	−18.3 ±3.8	−16.4 ± 3.1	−6.2 ± 2.2	−6.3 ± 2.4	−6.9 ± 2.5

**Fig. 4:**
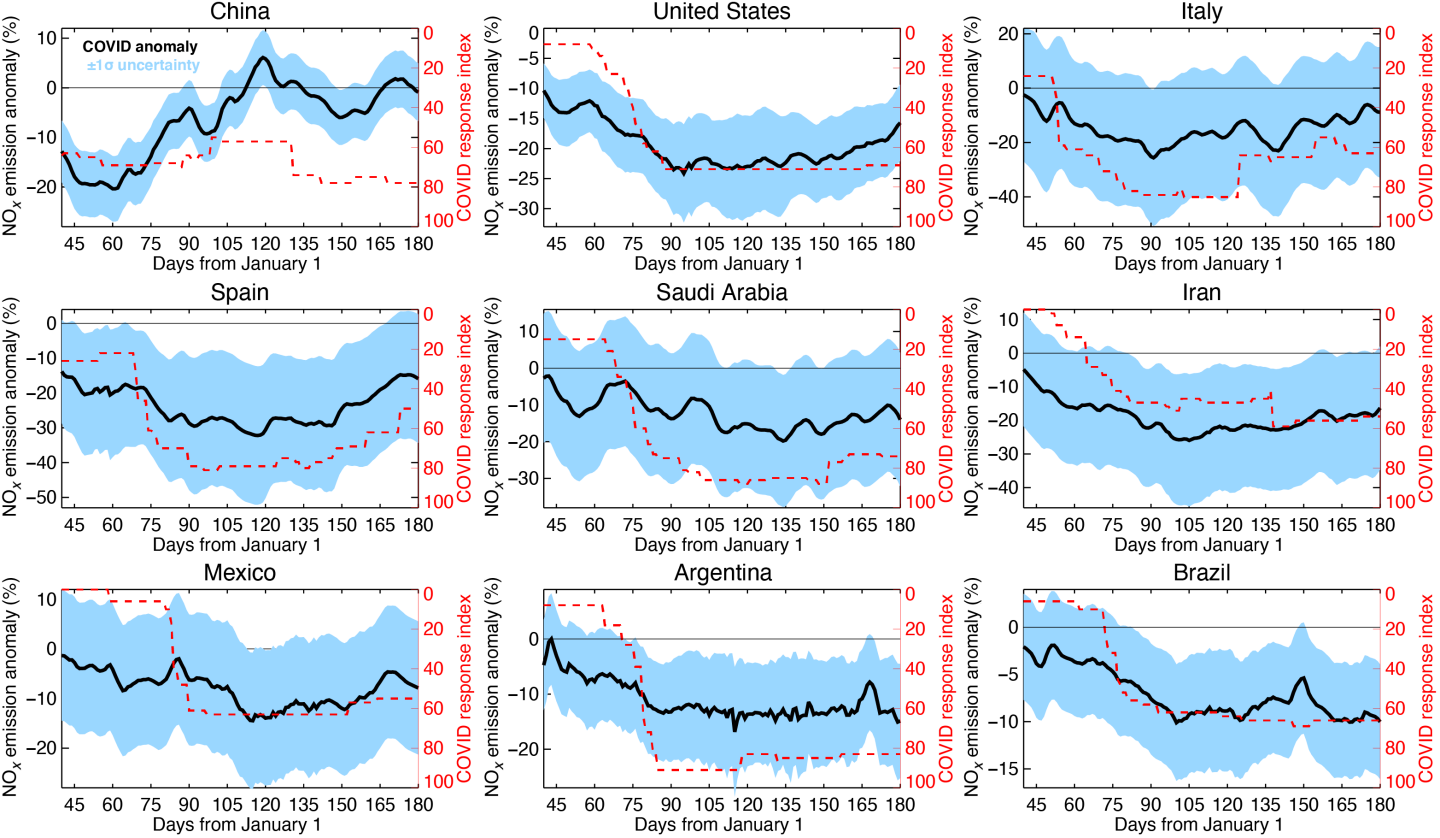
Time series of relative changes in country total NO_*x*_ emissions (in %, black line) due to the COVID-19 lockdowns. The COVID-19 Government Response Stringency Index is shown by the dashed red line. The *x* axis represents days from 1 January 2020. The shaded area represents the 1σ uncertainty as measured from the SD of the BAU emissions.

At the country scale, the estimated temporal evolution of anthropogenic emission reductions is strongly correlated with the COVID-19 Government Response Stringency Index (*23*), an indicator of the severity of government lockdown measures to slow transmission of COVID-19 (Fig. 4). The overall agreement between the NO_*x*_ emission reductions and the stringency index suggests that our emission analysis is able to capture the rapid changes in emissions linked to government actions globally (fig. S2), although the degree to which the two are correlated can be affected by compliance with the regulations and the relative importance of the transportation sector. Chinese NO_*x*_ emissions rapidly declined from late January through late February, corresponding to China’s first lockdown, followed by a rapid recovery to their normal levels for March and April. In May, the emissions again started to decrease, with a maximum reduction of 8% corresponding to a second lockdown in some parts of the country, such as Beijing, that was imposed to stop the second wave of COVID-19 cases. We estimate the maximum Chinese emission reduction to be 36% in total from early January to mid-February (*21*) and about 20% due to the COVID restriction when excluding the influence of the Chinese New Year holiday (Fig. 4). This estimated reduction is comparable to or slightly smaller than other top-down estimates using TROPOMI NO_2_: 20 to 50% reduction for most Chinese cities in February (*24*) and approximately 50% reductions during the lockdown (January 23 to February 9) relative to the period before the lockdown (*25*) and to the previous year (*26*). The subsequent recovery in March and rebound after early April are also common to other emission estimates (*24*, *26*). In Italy, the early implementation of lockdown led to large emission reductions, from late February to early May, of up to 25%. For other European countries such as France and Spain, both large emission reductions and high values of the Stringency Index are found from March through May. Most of the states in the United States announced emergency stay-at-home orders in late March. The estimated emissions show declines beginning in late February and early March, before the implementation of restrictive measures, with maximum reductions of about 25% in April and May, followed by a moderate recovery in June. These changes are broadly consistent with the Stringency Index (Fig. 4) and suggest that there was reduced traffic even before the stay-at-home order. However, there were cloudy conditions in February and March over some U.S. cities such as Los Angeles, which could have produced unstable emission corrections; this possibility will be explored further in a follow-up study. In Mexico, a nationwide lockdown was imposed in late March, and the NO_*x*_ emissions show a quick drop, with a maximum reduction of about 14% in April. Several Middle Eastern counties, such as Saudi Arabia and Iran, also show emission reductions of up to 25% from March through June, with a slight recovery in June. Limitations on human activity also affected emissions in South America. For instance, emissions from Brazil and Argentina were reduced by up to 10 and 15%, respectively, from March through June. The larger reductions in Argentina correspond to the stronger government response than in Brazil. A large emission reduction was also found over Lima, Peru (up to 30%) in April and May.

One of the confounding factors in attributing concentration changes to COVID-19–related emissions in tropical regions in Asia and Central Africa is biomass burning, which is often related to agricultural regions near more populated areas. To migrate these impacts, we use moderate resolution imaging spectroradiometer (MODIS) burned area data and outliner filtering (for model grids with rapid emission increases) to exclude biomass burning emissions (see Materials and Methods). Nevertheless, downwind regions may also be affected by enhanced NO_2_ concentrations linked to fires. In addition, possible errors in the model transport could lead to artificial adjustments to anthropogenic emissions in top-down estimates. The anthropogenic emissions around fire areas could be better estimated by combining our top-down emission estimates with in situ surface measurements and bottom-up inventories. Such an analysis, however, is left to future work.

Reductions in NO_2_ level during the lockdown have been evaluated at regional to country scales on the basis of observed concentration changes from previous years (e.g., 2019) to 2020. These reported changes are in reasonable agreement with our estimates, such as 23% reduction over Spain in April using TROPOMI (*27*) (in comparison to our COVID emission anomaly of 29%); up to 33% reductions over Tehran, Iran, using surface measurements (*28*) (26% over Iran in April in our estimates); 35% reductions over Almaty, Kazakhstan, using surface measurements (*29*) (27% over Kazakhstan in our estimates); 29 to 44% reductions over Istanbul, Turkey, using surface measurements (*30*) (24% in March in our estimates); and 15% reductions in March and 11% reductions in April over Greece using TROPOMI (*31*) (14% in March and 19% in April in our estimates). Nevertheless, the response of atmospheric composition to emission reductions needs to be considered in these comparisons. Using a machine learning technique and global surface measurements, the January to June mean global anthropogenic NO_*x*_ emission reduction was estimated at 3.1 (2.6 to 3.6) TgN (*32*), which is slightly smaller than the January to June average global emission reductions of 4.3 TgN in our study. Using TROPOMI NO_2_ measurements while accounting for solar angle and meteorological influences, NO_2_ decreases associated with the COVID-19 lockdowns were estimated at 9.2 to 43.4% for 20 cities in North America, with a median of 21.6% during March 15 and April 30 (*33*), which is comparable to our emission estimates (−16.1 ±4.3% in March and −20.7 ±6.2% in April for North America).

The estimated anthropogenic NO_*x*_ emission changes that we show here are broadly consistent with those based on bottom-up emission estimates for the COVID-19 period (*3*–*5*). Nevertheless, the NO_*x*_ emission estimates based on activity data (*4*) suggest larger global total emission reductions (about 30% in April) than our estimates (14.8 ±2.3% relative to the globe total anthropogenic emissions and 21± 3.3% for the analyzed area), with larger contributions from China (about 2.5% of the global total emission reduction, in contrast to 1.0% in our estimate) and smaller contributions from Europe (about 2%, in contrast to 4%). Although the temporal changes in NO_*x*_ are generally consistent for major polluted countries, the bottom-up estimates indicate larger reductions in NO_*x*_, for instance, up to 40% for the United States (in contrast to 24% relative to the global total emissions and 34% relative to the analyzed area total emissions for our top-down estimates), 57% for Italy (25 and 32%), 64% for Spain (32 and 34%), 54% for Saudi Arabia (20 and 34%), 49% for Mexico (14 and 32%), 52% for Argentina (17 and 45%), and 43% for Brazil (17 and 32%). These discrepancies could reflect large uncertainties in the activity data, which is limited to selected sectors, used in bottom-up estimates. In contrast, our top-down approach infers total emission changes. In addition, while top-down emissions offer great potential to supplement or improve bottom-up inventories, they also contain large uncertainties associated with errors in chemical transport modeling and assimilated observations (*34*, *35*).

Although the multiconstituent data assimilation provides comprehensive constraints on the tropospheric chemistry system, remaining model errors, for instance, in planetary boundary layer mixing, convective transport, lightning NO_*x*_ sources, and NO_*x*_ chemical lifetime (*36*) can lead to artificial adjustments and biased emission estimates. In addition, because our NO_*x*_ emissions rely on TROPOMI NO_2_ observations at its overpass time (13:30 local time), any diurnal variation of the emission reductions (e.g., a larger reduction during morning and evening rush hours than in the middle of the day) could explain part of the discrepancy. The TROPOMI NO2 tropospheric column data show a negative bias of typically −23 to −37% in clean to slightly polluted conditions and −51% over highly polluted areas on average compared to ground-based measurements (*22*). The difference with ground-based observations scales roughly linearly with the tropospheric NO_2_ column. This bias has been partly attributed to a negative bias in the cloud height in FRESCO (fast retrieval scheme for clouds from the oxygen A band) and affected the country-scale emission analysis. Nevertheless, these systematic errors likely change only slightly before and after the COVID lockdown and likely not substantially affect the magnitude and temporal evolution of the COVID emission anomaly. Aerosols can also have an impact on the NO_2_ retrievals, depending on the type of aerosol and distribution. The OMI-quality assurance for essential climate variables (QA4ECV) and TROPOMI retrievals implicitly account for the amount of aerosol scattering and the altitude in the atmosphere where the scattering takes place through the effective cloud pressure and fraction derived from the TROPOMI observations. The influence of aerosols on TROPOMI NO_2_ biases could vary before and during the lockdowns, which needs to be explored and addressed in future retrieval work. Furthermore, if the a priori inventories mislocate emission sources, then optimizing scaling factors as in this study could lead to underestimation of the total emission reductions. Meanwhile, an important advantage of our top-down estimate is the consistent global analysis, whereas bottom-up inventories suffer from regional dependencies with respect to the accuracy of activity data. By comparing COVID emission anomaly estimates from different approaches, improved estimates of their uncertainties could be obtained. Further detailed comparisons of spatial and temporal emission patterns between the top-down and bottom-up estimates and additional quantification of top-down uncertainty will play an essential role in the future exploration of the COVID emission anomaly.

### Tropospheric ozone response

Using the BAU emissions and 2020 emissions with the same meteorological conditions allows us to evaluate tropospheric ozone concentration changes directly linked to COVID-19 emission declines while accounting for the “observed” meteorology (as filtered through a reanalysis system; see Materials and Methods). This approach is in contrast to studies that evaluate atmospheric composition anomalies in 2020 directly from comparisons between 2020 conditions and previous years (*7*, *11*). In these studies, the confounding factors of meteorological variations and spatiotemporal differences in the relationship between atmospheric concentrations and emissions add substantial, but poorly constrained, uncertainty in their inferences of COVID-19 effects on atmospheric composition.

**Fig. 5:**
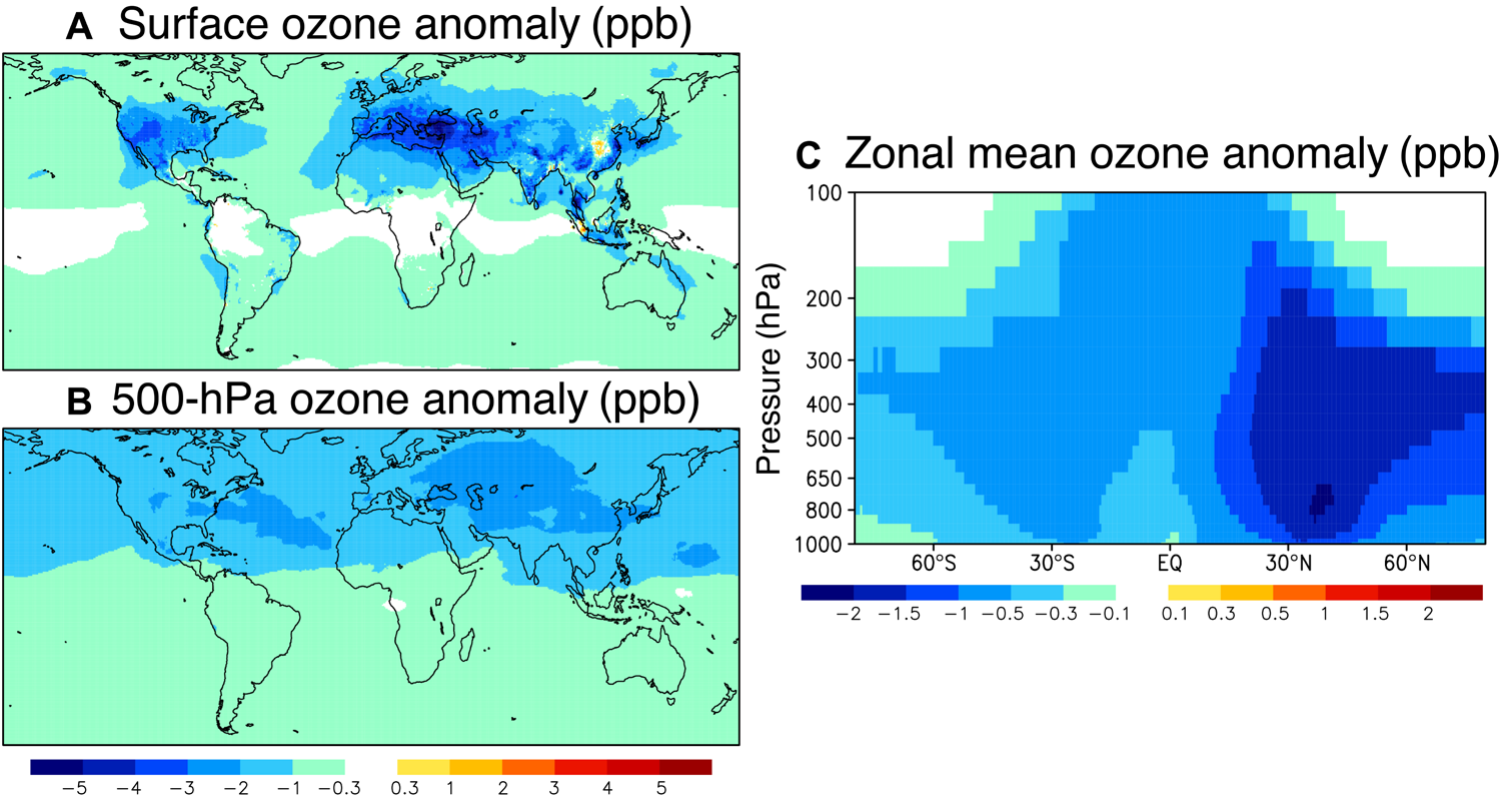
Monthly ozone changes due to the COVID NO_*x*_ emission reductions in May 2020. Spatial distribution of the ozone anomaly (in ppb) at (**A**) the surface, (**B**) 500 hPa, and (**C**) zonal mean values in latitude-pressure coordinates. EQ, equator.

**Fig. 6:**
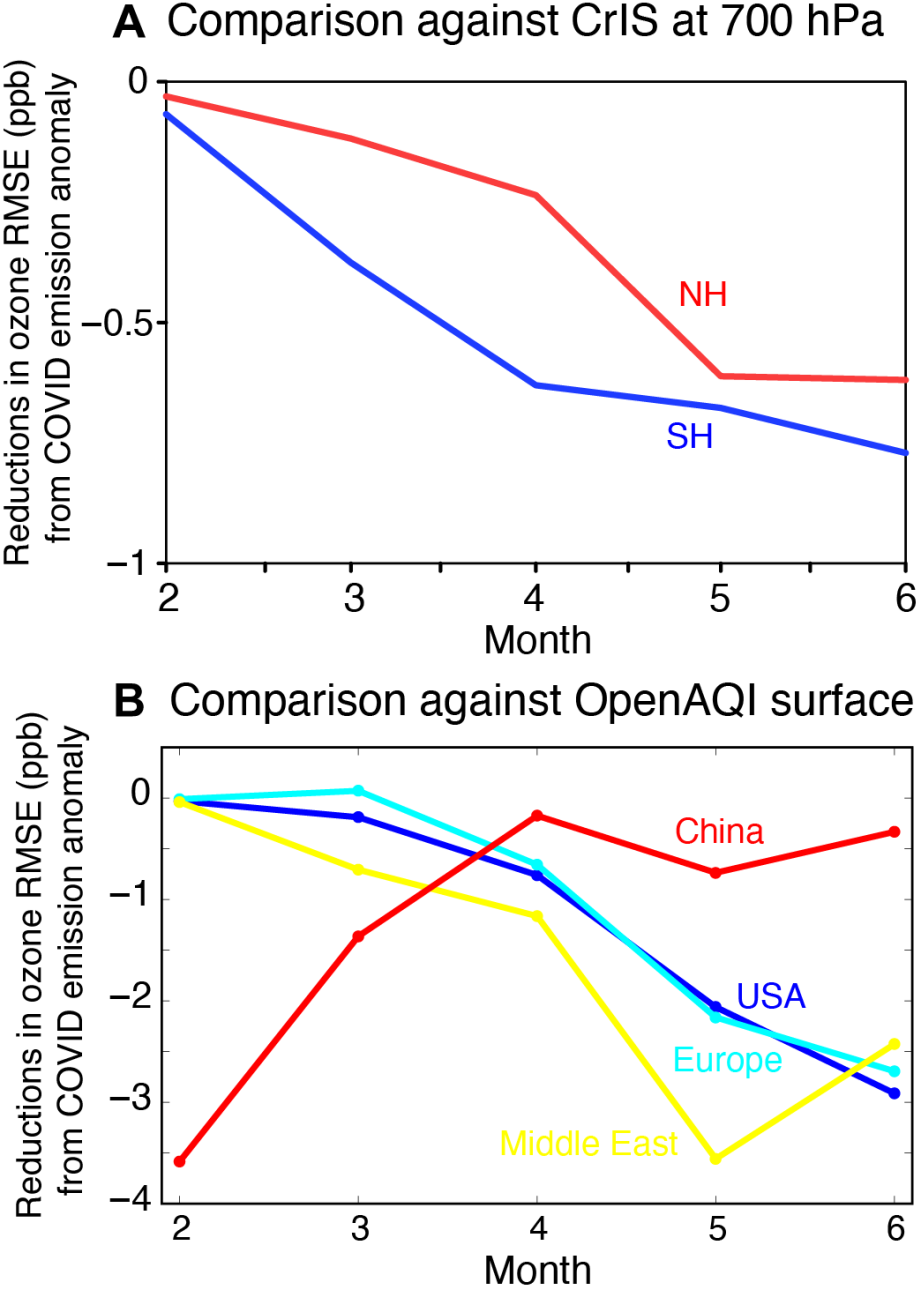
Comparisons to ozone measurements from the CrIS satellite and surface networks. Time series of differences in monthly root mean square errors (RMSEs) (in ppb) of ozone against (**A**) the CrIS satellite retrievals at 700 hPa for the Northern Hemisphere (NH) (20°N to 90^∘^N) and Southern Hemisphere (SH) (90°S to 20^∘^S) and (**B**) the surface observations from the OpenAQ platform for Europe (light blue), the United States (blue), the Middle East (yellow), and China (red). The RMSE differences were estimated from two model simulations using the BAU and 2020 emissions, where the negative values show improved agreement against the observations using the 2020 emissions.

Our sensitivity simulations show a strong response of ozone to the COVID-19 NO_*x*_ reductions that extends from the surface to the upper troposphere (Fig. 5). The results using our 2020 emissions show better agreement with observed concentrations from in situ measurements from February through June except over Europe in February and March and over the Middle East in February. The 2020 emission simulation also provides better agreement with ozone retrievals from the Cross-Track Infrared Sounder (CrIS) satellite instrument (*37*) than that using BAU emissions for the area mean ozone concentrations at 700 hPa over China, the United States, Europe, and Middle East from February through June except over Europe in February and March (Fig. 6 and fig. S3). These ozone measurements were not used in data assimilation. At the local scale, and especially near the surface, the estimated ozone response varies greatly with location and time as a consequence of differences in photochemical regime, which depends on a number of factors other than NO_*x*_. These factors include the amount and reactivity of VOCs (climatological VOC emissions were used in all simulations; see Materials and Methods), background oxidant levels, and meteorological conditions. Over highly polluted urban areas with high NO_*x*_ concentrations, NO_*x*_ reduction can enhance ozone production due to NO_*x*_ titration; this response is mainly due to enhanced atmospheric oxidation capacity in these locations, which is reflected in the levels of major oxidants [e.g., the hydroxyl radical (OH) and nitrate radical (NO_3_)] (*38*). Therefore, NO_*x*_ emission reductions can increase ozone locally over urban areas because of higher levels of OH and reactions with VOCs. Increased surface ozone was seen in our estimates over parts of northern Europe, China, and South Africa, as has already been reported for northern China during the lockdown (*10*, *21*). Nevertheless, the obtained ozone production efficiency (OPE, mass of ozone produced per unit mass of NO_*x*_ emitted) for the monthly mean tropospheric ozone burden (TOB, in TgO_3_ unit, integrated from the surface to the tropopause globally) based on the regional emission changes was mostly positive throughout the analysis period (i.e., NO_*x*_ emission declines reduced TOB), as seen in other modeling studies (*39*).

The globally averaged tropospheric lifetime of ozone is relatively short (23 days) (*40*). Therefore, the influence of NO_*x*_ emission reductions on TOB can be accumulated during the course of the COVID pandemic. Thus, we evaluated cumulative total tropospheric ozone changes from model simulations starting in February 2020. As summarized in Fig. 3B, the estimated ozone response shows substantial seasonal variations as a consequence of varying meteorological and chemical conditions in addition to emission changes. In total, the global TOB decreased by 0.6 TgO_3_ in February and by 6.5 TgO_3_ in June, reflecting an order of magnitude intensification in the decline in just over 5 months. The reduced ozone associated with the COVID-19 emissions accounted for about 2% of TOB (∼300 TgO_3_) in May and June. Because areas that account for about 25% of the global total anthropogenic NO_*x*_ emissions were removed from our estimates, including tropical biomass areas with strong OPE, the actual ozone changes may be even larger. Assuming that the removed areas had similar relative emission reductions as the surrounding areas, we obtain a reduction of up to 9 TgO_3_, about 3% of TOB. The rapid decreases in emissions and consequent concentrations can be thought of as going either backward in time to former anthropogenic emission levels or forward in time to a set of emission targets if these emissions were sustained over longer periods of time (*41*). By comparison, the most aggressive representative concentration pathway (RCP) defined for the Climate Model Intercomparison Project-5 (RCP 2.6) projects a reduction of TOB of about 4% by 2030 (*40*). Applying the average satellite-derived TOB trend over the past two decades (+0.71 TgO_3_/year, from −2.15 to +2.85 TgO_3_/year for different satellite sensors) (*42*), the COVID-19 TOB reductions of 6 to 9 TgO_3_ are equivalent to going back in time to TOB values for 2007–2011. These TOB reductions correspond to a tropospheric ozone radiative forcing of 233 to 350 mWm^−2^ based on the global mean normalized tropospheric ozone radiative forcing of 42 mWm^−2^DU^−1^ from the Atmospheric Chemistry and Climate Model Intercomparison Project (ACCMIP) simulation results (*43*).

**Fig. 7:**
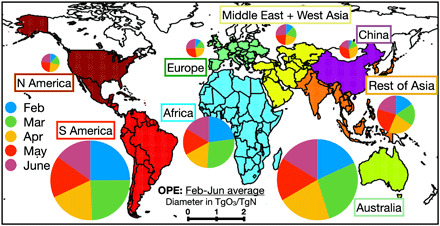
Global map of the OPE. OPE is estimated from the change in the global TOB corresponding to the regional COVID NO_*x*_ emission anomaly. The diameter of each circle represents the averaged OPE value during February to July 2020 in TgO_3_/TgN, while each sector of the circle represents the relative OPE magnitude for each month. The background map shows the defined areas used in this study.

To identify regional and seasonal changes in the ozone response, we conducted sensitivity calculations using BAU and 2020 emissions. The impacts were measured by comparing simulation results from a control model simulation using the BAU NO_*x*_ emissions and sensitivity model simulations using 2020 emissions for the region of interest and BAU emissions everywhere else (cf. Fig. 7). The contributions of emissions from each region to TOB varied substantially with time. In February, the large emission reductions in China had little impact on ozone. In March, Asia (including China) and South America account for about 60% of the total ozone reduction (2.5 TgO_3_). In April, the total reduction of 4.7 TgO_3_ is mainly attributed to emission reductions in Asia, China, North America, and South America (0.7 to 0.8 TgO_3_ for each region). In May and June, when the reduction in TOB reached its maximum value, the emissions from the rest of Asia (excluding China, whose emissions had largely recovered by that time) have the largest contribution to the total ozone reduction (1.2 to 1.5 TgO_3_ of 6.0 to 6.5 TgO_3_), followed by North America (1.2 TgO_3_) and South America (0.8 TgO_3_). The NO_*x*_ emissions from the Middle East and West Asia, Europe, Africa, and Australia provided minor contributions to the global ozone budget from February through June.

The ozone reductions corresponding to the emission decreases in each region exhibit distinct spatial patterns, including both local and remote impacts (Fig. 5 and fig. S6). For instance, free tropospheric ozone over Eastern and Central Eurasia is reduced because of North American emission reductions, whereas the South American emission reductions result in a long tail of decreased ozone along the mid-latitude westerlies in the Southern Hemisphere (SH). In all, the COVID-19 NO_*x*_ reductions led to up to 10–parts per billion (ppb) reductions at grid scale (in southern Malaysia) and about 5-ppb reductions at regional scale in monthly mean ozone at the surface and 3-ppb reductions at 500 hPa (Fig. 5, A and B). In terms of vertical propagation, the European and Australian emission influences on ozone are mostly limited to the region below 300 hPa and poleward of 30°. These patterns are likely dominated by quasi-isentropic transport linked to mid-latitude synoptic-scale disturbances. The ozone anomalies from the Middle Eastern and West Asian, South American, and North American emissions extend up to 200 hPa in the subtropics through deep convection, with up to 1-ppb reductions in the monthly and zonal mean concentration in the upper troposphere (Fig. 5C and fig. S7). Asian emissions show a distinct pattern, with maximum values of the ozone anomaly in the upper troposphere and the anomaly extending throughout the tropics to the mid latitudes of both hemispheres. This pattern reflects not only convection over the maritime continent but also transport through the Asian monsoon, suggesting substantial impacts of Asian human activity on the global environment. The latitudinal and vertical propagation of ozone anomalies seen in Fig. 5 and figs. S6 and S7, with 2 to 5% reductions in the zonal mean concentration in the tropics and Northern Hemisphere (NH) subtropics and 1 to 2% reductions in the SH and NH extratropics, signifies important implications for ozone radiative forcing, as ozone has the largest impact on the top-of-atmosphere flux in the middle and upper troposphere (*44*). The NO_*x*_ reductions could also affect radiative forcing through decreases in nitrate aerosol; the impacts on secondary aerosol formation need to be further addressed in a future study.

Reduced NO_*x*_ emissions during the COVID period also led to decreases in free tropospheric peroxyacetyl nitrate (PAN) concentrations over both polluted regions [by up to about 35 parts per thousand (ppt)] and remote areas such as northern and southern Atlantic and Pacific oceans (by up to about 10 ppt; fig. S8B). PAN is a long-lived reservoir species for NO_*x*_ and can be transported long distances from source regions before decomposing; these results highlight the nonlocal impacts of the emission reductions on global ozone through long-range transport of precursors. Meanwhile, substantial reductions in grid-scale local tropospheric mean OH of up to 30% locally (fig. S8A) suggest substantial impacts of the worldwide lockdowns on the entire tropospheric chemistry system, including on the chemical lifetimes of many species such as methane. The maximum reduction of the tropospheric global mean OH concentration, which occurs in May, is 4.0%. Using the ACCMIP multimodel mean estimate of the tropospheric chemical methane lifetime (9.3 ± 1.6 years) (*45*), the 4.0% OH reduction would increase the methane lifetime by about 4 months.

The OPE was estimated using the global TOB response corresponding to reduced NO_*x*_ emissions for each region of the world and for each month separately. The OPE increased by a factor of 2 to 5 from February to July in the NH mid and high latitudes, as illustrated in Fig. 7 and described in Table 2, largely because of the increasing availability of sunlight from the winter to the summer season. When averaged over the February to June time period, tropical and SH low- and mid-latitude regions, such as Africa, South America, and Australia, show much larger OPE values (1.9 to 2.9 TgO_3_/TgN) than those in the NH extratropics (0.2 to 0.4 TgO_3_/TgN). The OPE variations help to explain the different ozone response patterns described above. The impact of the large extratropical NH NO_*x*_ emission reductions on tropospheric ozone is relatively small because of the weak OPE, especially in the winter and spring seasons. In contrast, ozone reductions are much larger for tropical regions such as South America, despite smaller NO_*x*_ changes, because of the larger OPE. These results suggest that considering where and when government actions to slow the spread of COVID-19 occurred is extremely important in understanding the impacts of COVID lockdowns on atmospheric composition.

**Table 2:** Monthly values of the regional OPE (in TgO_3_/TgN). The OPE was estimated for the global TOB, using the regional COVID-19 NO_*x*_ emission anomalies. The 1-sigma uncertainties, estimated from the SD (i.e., temporal changes) of the estimated TOB during the analysis period, are also shown.

**Region**	**February**	**March**	**April**	**May**	**June**
Africa	2.15 ± 0.08	2.61 ± 0.23	1.51 ± 0.17	1.56 ± 0.08	1.53 ± 0.12
Europe	0.09 ± 0.01	0.13 ± 0.01	0.20 ± 0.06	0.23 ± 0.03	0.23 ± 0.03
Australia	2.68 ± 0.10	4.01 ± 0.10	3.16 ± 0.04	2.54 ± 0.05	2.40 ± 0.08
Middle East + W Asia	0.25 ± 0.01	0.40 ± 0.05	0.45 ± 0.05	0.47 ± 0.05	0.57 ± 0.05
Rest of Asia	1.11 ± 0.04	1.35 ± 0.03	1.54 ± 0.15	1.65 ± 0.07	1.44 ± 0.04
S America	3.65 ± 0.11	3.55 ± 0.09	2.75 ± 0.04	2.47 ± 0.04	2.21 ± 0.04
N America	0.23 ± 0.01	0.33 ± 0.04	0.45 ± 0.08	0.50 ± 0.06	0.45 ± 0.06
China	0.08 ± 0.00	0.17 ± 0.01	0.37 ± 0.05	0.44 ± 0.04	0.25 ± 0.02

The response of ozone to changes in NO_*x*_ emissions can differ substantially between chemical transport models. In our previous work using a multimodel chemical data assimilation system (*46*), we obtained up to a factor of 2 difference in surface ozone response among different models due to fundamental differences in the representation of fast chemical and dynamical processes. At the same time, multimodel intercomparison studies have demonstrated that the ozone response to varying NO_*x*_ emissions in the Model for Interdisciplinary Research on Climate (MIROC)–chemical atmospheric general circulation model for study of atmospheric environment and radiative forcing (CHASER) model (*47*) used here fits well within the multimodel estimates (*43*, *48*, *49*).

**Fig. 8:**
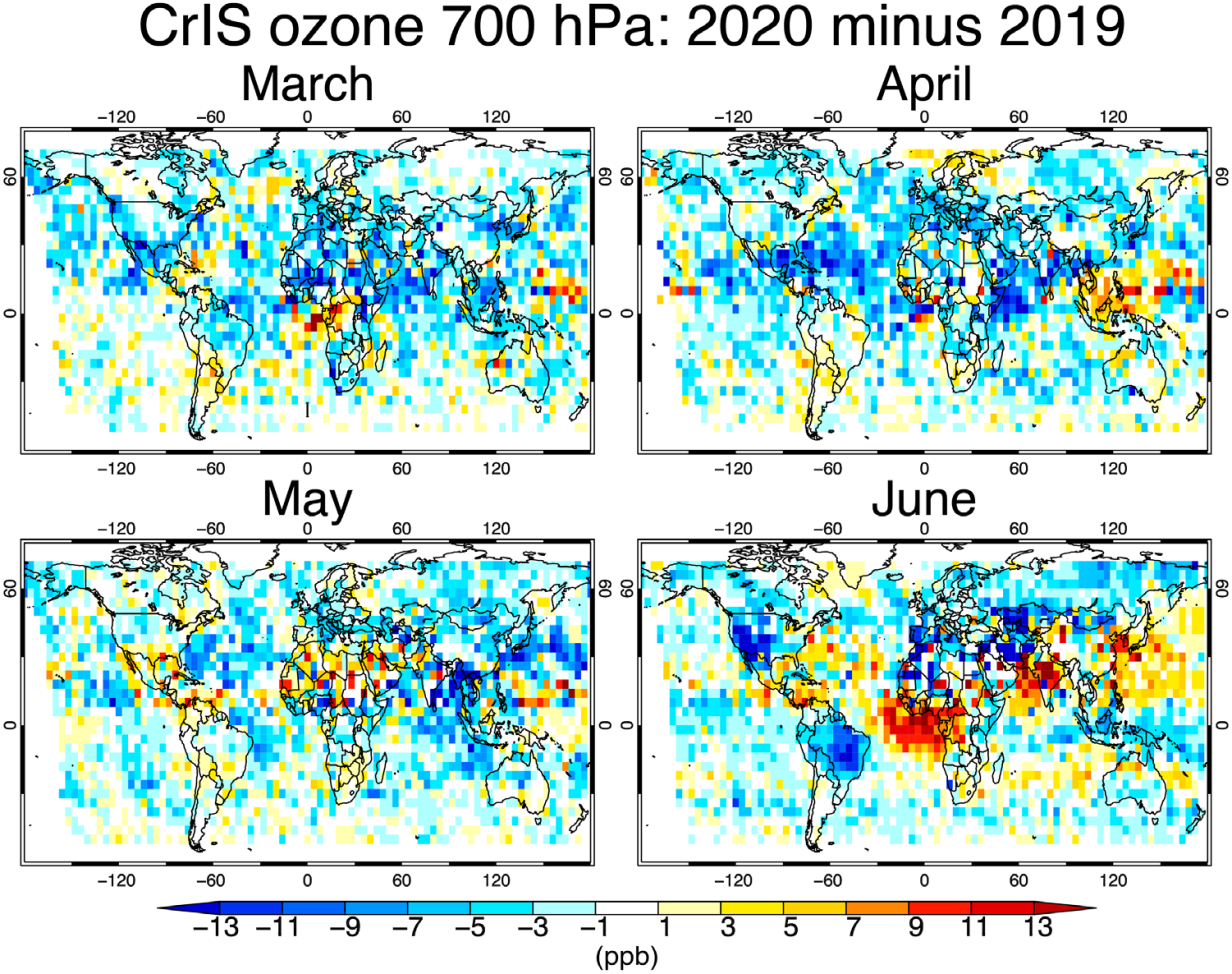
Monthly ozone changes observed from CrIS from 2019 to 2020. Global distributions of monthly mean ozone concentration difference between 2020 and 2019 (2020 minus 2019) observed from the CrIS satellite measurements at 700 hPa for March to June 2020. Negative values (blue) represent lower ozone concentrations in 2020 than in 2019.

Our modeled ozone responses to COVID-19 NO_*x*_ emissions are broadly consistent with observed ozone changes. Recently developed tropospheric ozone profile retrievals from the CrIS satellite instrument (*37*) provide an opportunity to evaluate the simulated ozone responses. The modeled ozone in the free troposphere shows closer agreement with the CrIS observations for many regions when using the 2020 emissions than when using BAU emissions (Fig. 6A). The discrepancy between CrIS and the BAU emission scenario increases from April through June. The root mean square error (RMSE) reduction associated with the COVID emissions reaches 20% in May and June, while the mean bias against the CrIS data in June is reduced by using the COVID emissions from 3.2 to 2.0 ppb in the SH and from 2.0 to 1.1 ppb in the NH. The improvements in bias and RMSE are furthermore large over the regions most affected by COVID, with the exception of Africa, where the 95% improvement in bias may be due to greatly underestimated a priori emissions (fig. S3). The CrIS observations also show clear reductions in the free tropospheric ozone from 2019 to 2020 by 1 to 12 ppb over most of the polluted areas (Fig. 8), with reductions of zonal mean concentrations by up to 4 ppb at NH mid latitudes from March through June. These observed changes are driven by various factors including emissions, meteorology, and biomass burning. Nevertheless, the broadly consistent results between the observed changes from 2019 to 2020 and the simulated ozone response to the reduced NO_*x*_ emissions in 2020 (Fig. 5) suggest that parts of the influence of the COVID emission anomaly are observable in the free troposphere. At the surface, the RMSE between the model and measurements is reduced by 20 to 40% for Europe, the United States, and China when considering the COVID-19 emission reductions (Fig. 6B, figs. S4 and S5, and table S1). The observed surface ozone changes between 2020 and previous years show complex patterns across the globe (*11*, *50*), reflecting changes in local meteorology and urban nonlinear chemistry as well as in emissions (*13*). Further investigation with a focus on surface local air quality is required to understand how the observed changes in surface ozone are related to the COVID-19 emission anomaly and is beyond the scope of this study. Meanwhile, the lack of emission changes other than NO_*x*_, such as VOCs and CO, in the model simulations could explain part of the discrepancy (Fig. 6B, figs. S4 and S5, and table S1). Additional model evaluation results are provided in the Supplementary Materials (sections S1 to S4).

Here, we have shown the impacts of COVID-related NO_*x*_ emission reductions on global tropospheric ozone, but there are additional considerations that should be investigated further to fully understand the implications of COVID-19 emission changes for ozone. For example, inconsistencies in TROPOMI sampling, mainly due to clouds, may have affected the estimated short-term variations in NO_*x*_ emissions. Furthermore, although the model used has a relatively high spatial resolution for the globe (0.56°), the simulation of surface concentrations is sensitive to model resolution, owing to the fine-scale distribution of emissions and transport as well as resolution-dependent nonlinear effects in the NO_2_ loss rate (*51*). Aerosol levels were also greatly affected by COVID-19 (*52*), which may have had an additional impact on ozone chemistry. Simultaneous reductions in primary aerosol and NO_*x*_ emissions could have the effect of increasing ozone (*19*). The absence of changes in primary aerosol in our COVID emission estimates might explain some of the remaining model ozone bias, especially at the surface. In addition, the contribution of VOCs to ozone changes during the COVID period is essentially unknown because of the lack of either bottom-up or top-down emission estimates for the period. Nevertheless, if we add a global reduction in anthropogenic VOC emissions of 20% to our 2020 emission scenario while also considering the COVID-19 NO_*x*_ emission reductions, then we find additional reductions in monthly mean surface ozone of up to 3 ppb over eastern China and less than 1 ppb over the rest of the NH; 500-hPa ozone is reduced by less than 1 ppb globally in May 2020 (fig. S9, A and B). The accumulated influence of the 20% VOC emission reductions on the global TOB reaches 1.6 TgO_3_ in June 2020, leading to an additional 22 to 26% reduction in monthly global TOB for March to June 2020 (fig. S9C). Our ozone anomaly estimates based on NO_*x*_ emission reductions alone may thus underestimate the actual TOB reductions. In addition, decreased CO concentrations during the lockdowns were reported using in situ and satellite observations (*53*–*55*), which could also suppress photochemical ozone productions. Nevertheless, because of its relatively long chemical lifetime and the influences of long-range transports, detailed distributions of CO emission reductions during the lockdowns have not yet been illustrated and not considered in our estimates. In many cases, changes in CO emissions have much smaller impacts on ozone than those in NO_*x*_ emissions (*56*). A more detailed analysis of the contribution of changes in VOCs and CO needs to be addressed in future work, along with a means to validate the results.

## DISCUSSION

The worldwide actions taken to slow the transmission of COVID-19 had the effect of rapid emission reductions globally, which drove substantial changes in air pollutants and tropospheric chemistry. The pandemic took place against a backdrop in which many countries have implemented environmental policies to reduce human health risk from air pollution by controlling emissions, but the quantitative impacts of these policies have not always been clear (*57*–*59*). COVID-19 represents a well-observed “scenario-of-opportunity” that allows us to assess how atmospheric composition responds to reduced human activity and emissions, providing an important benchmark for identifying effective environmental policy-making. Here, we have evaluated global NO_*x*_ emission reductions and their impacts on global tropospheric ozone, using a state-of-the-art multiconstituent data assimilation system.

The COVID-19 restrictions on human activity in numerous countries led to substantial reductions in global total anthropogenic NO_*x*_ emissions of at least 15% in April and May 2020, with 19 to 25% reductions in the United States, Europe, and the Middle East and West Asia. Using the estimated emission reductions, we find that the tropospheric ozone response to the NO_*x*_ emission reductions exhibited strong spatial and temporal gradients as a consequence of differences in OPE, with larger values in the tropics and SH subtropics (1.9 to 2.9 TgO_3_/TgN, February to June average) than in the NH mid and high latitudes (0.2 to 0.4 TgO_3_/TgN). The OPE in the NH extratropics increased by a factor of 2 to 3 from February to June. The reduction in ozone associated with COVID-19 changes in NO_*x*_ is as large as 10 ppb and is seen both at the surface and in free tropospheric concentrations. The COVID-related ozone anomaly is widespread in the NH and is substantial even in the SH, especially downwind of megacities in South America. Overall, the pandemic led to a modeled 6-TgO_3_ (∼2%) decrease in TOB in May and June. Decreased concentrations of PAN and OH suggest highly nonlocal impacts of the lockdowns and substantial changes in the tropospheric chemistry system.

The results described here demonstrate the strong impacts of the worldwide restrictions on human activity on global tropospheric chemistry and radiative forcing. Our model indicates rapid reductions in free tropospheric ozone concentrations of up to 5 ppb in March to June 2020 over major polluted areas and is in broad agreement with the CrIS satellite observations. Our study thus benefits future predictions of the chemistry-climate system by providing validation of our understanding of the response of tropospheric ozone to changes in NO_*x*_ emissions.

Our results indicate that the designers of environmental policies to benefit both air quality and climate need to carefully consider the complex relationships between emissions and atmospheric composition such as those demonstrated here to effectively improve air quality and reduce radiative forcing, especially for countries in the tropics that have a combination of high population density and large OPE. However, we focused here on regional and global ozone responses to NO_*x*_ emission changes and the potential impacts of VOC emission changes; urban-scale emission changes and subsequent ozone responses were not well resolved. Further investigation of NO_*x*_/VOC impacts at finer (e.g., urban) scales is essential to assessing the implications of the COVID-19 lockdowns on air quality and human health. Last, our ozone response estimates for the COVID-19 pandemic provide insights into where and when the atmospheric composition effects of the pandemic may be measurable directly from observations.

## MATERIALS AND METHODS

### Experimental design

The surface NO_*x*_ emission reductions associated with the COVID-19 lockdowns were estimated using a top-down approach within a state-of-the data assimilation system (*20*). The obtained emission reductions were used to evaluate the tropospheric ozone response and OPE for each region of the world using the MIROC-CHASER global chemical transport model (CTM).

### Top-down surface NO_*x*_ emission estimates

An updated version of the Tropospheric Chemistry Reanalysis version 2 (TCR-2) (*20*) is used to evaluate NO_*x*_ emission changes and their influence on ozone concentrations. The TCR-2 dataset is available at https://doi.org/10.25966/9qgv-fe81. The reanalysis is produced via the assimilation of multiple satellite measurements of ozone, CO, NO_2_, HNO_3_, and SO_2_. The tropospheric NO_2_ column retrievals from the QA4ECV version 1.1 level 2 product for OMI (*60*) and TM5-MP-DOMINO version 1.2 for TROPOMI (*61*) were used to constrain NO_*x*_ emissions. We used a super-observation approach (*62*) to generate representative data with a horizontal resolution of the forecast model. The OMI SO_2_ data used were the planetary boundary layer vertical column SO_2_ level 2 (L2) product obtained with the principal components analysis algorithm (*63*). The MOPITT (measurements of pollution in the troposphere) total column CO data used were the version 7 L2 thermal infrared/near-infrared product (*64*). Version 4.2 ozone and HNO_3_ L2 products from Microwave Limb Sounder (*65*) were used to constrain the chemical concentrations in the upper troposphere and lower stratosphere. The model and data assimilation calculations for 2020 in this study were conducted at 0.56° horizontal resolution using the MIROC-CHASER and an ensemble Kalman filter technique that optimizes both chemical concentrations of various species and emissions of NO_*x*_, CO, and SO_2_.

The emission estimation is based on a state augmentation technique, which has been used in our previous studies (*34*, *46*, *56*, *62*, *66*, *67*). This approach allows us to reflect temporal and geographical variations in transport and chemical reactions in the emission estimates. The emissions in the state vector are represented by scaling factors for each surface grid cell. Thus, emission sources that are mislocated or not represented by the a priori emissions cannot be adjusted by data assimilation. Only the combined total emission is optimized in data assimilation, where the ratio of different emission categories within the a priori emissions for each grid point was applied to the estimated emissions after data assimilation to obtain the a posteriori anthropogenic emissions. The quality of the reanalysis fields for 2005–2018 has been evaluated on the basis of comparisons against ozonesondes and independent aircraft and satellite observations for various chemical species on regional and global scales, as well as for seasonal, yearly, and decadal scales, from the surface to the lower stratosphere (*20*). The emissions for 2020 constrained by TROPOMI NO_2_ at 0.56° horizontal resolution have already been used to evaluate the air quality response to the Chinese COVID-19 lockdown (*21*).

To evaluate emission anomalies due to the COVID-19 restrictions, the influence of climatological temporal emission variations and interannual changes from previous years to 2020 was removed by comparing the 2020 optimized emissions with the baseline BAU emissions constructed on the basis of our decadal chemical reanalysis, which is constrained by OMI NO_2_ (*20*). The following steps were taken to obtain the BAU emissions for 2020 at each grid point, as illustrated in Fig. 1. (1) The 2010–2019 emissions obtained from the reanalysis were used to evaluate relative temporal emission changes from February 1 (January 10 for China only) through July 31 each year. (2) The calculated relative temporal emission changes were averaged over the 10 years (2010–2019) to obtain climatological relative emission variations. (3) The climatological relative emission variations were applied to the 2020 emission values on 1 February 2020 (10 January 2020 for China) through 31 July 2020 to obtain the BAU emissions for 2020. Because the emissions changed only gradually during the non-COVID periods in the reanalysis, the choice of the base date did not substantially affect the estimated COVID emission anomaly. While the emission estimates based on long-term OMI records enabled us to evaluate climatological emission variations, assimilation of TROPOMI NO_2_ for 2020 provided strong constraints on the detailed spatiotemporal variations in the 2020 COVID-19 emissions, as confirmed by consistent variations with the assimilated NO_2_ measurements (fig. S1) and surface measurements (table S1). The influences of systematic biases between TROPOMI and OMI measurements, along with the influences of interannual changes in emissions, were excluded by aggregating the normalized temporal variability for each year.

On the basis of the comparisons between the 2020 and BAU emissions, we estimated the COVID emission anomaly, which eliminates the impacts of the climatological seasonal changes in emissions, such as the use of wintertime heating and enhanced soil emissions in summer, as well as interannual variations. In addition, in top-down estimates, systematic model errors, for instance in the seasonally varying chemical lifetime of NO_*x*_, can cause artificial seasonal changes in emissions, which are also removed by comparing the BAU and 2020 emissions constructed using the same system. Biomass burning signals in emissions were removed using MODIS burned area information (*68*) for 2020. In addition, we used climatological monthly FINN (fire inventory) emissions averaged over 2010–2019 (*69*) and removed grid points with average FINN emissions larger than 2×10^−12^ kg Nm^−2^ s^−1^ where the BAU emissions were likely affected by fires. Countries in Central America, northern South America, and Central Africa were largely affected by these fire filtering. We also removed those grid points that showed rapid emission increases, defined as 3-day averaged emissions during the lockdown period that were two times larger than the February to June averaged emissions.

Because of the relatively large uncertainty and limited coverage of the assimilated measurements, grid points poleward of 55° in both hemispheres and countries including those grid points (Canada, Russia, and northern Europe, except for the United Kingdom), as well as ocean grid points (i.e., ship emissions), were also excluded from the analysis. Areas that were heavily affected by clouds, as measured from variability of emission increments during the analysis period, were also removed from data assimilation analysis. In total, areas with about 25% of the global total NO_*x*_ emissions were excluded from our analysis. For China, the impact of the Chinese New Year holiday was removed from the 2020 emissions to separately evaluate the COVID-19 anomaly using the baseline emission variations relative to the Chinese New Year date each year, following the method in our previous study (*21*).

### Chemical transport model, MIROC-CHASER

The forecast model used in the chemical data assimilation and sensitivity model calculations is MIROC-Chem (*47*, *70*) at 0.56° horizontal resolution. The model simulates spatial and temporal variations in chemical species in the troposphere and stratosphere by calculating tracer transport (advection, cumulus convection, and vertical diffusion), emissions, dry and wet deposition, and chemical processes (92 species and 262 reactions) including the ozone-HO_*x*_-NO_*x*_-CH_4_-CO system with nonmethane VOC oxidation. It also includes stratospheric chemistry such as halogen chemistry. Lightning NO_*x*_ sources were calculated in conjunction with the convection scheme of MIROC–atmospheric general circulation model (AGCM). The meteorological fields were calculated using the MIROC-AGCM (*47*). The simulated meteorological fields were nudged to the six-hourly ERA-Interim reanalysis data (*71*). For data assimilation calculations, the a priori anthropogenic emissions of NO_*x*_, CO, and SO_2_ were obtained from HTAP (hemispheric transport air pollution) version 2 for 2010 (*72*), which were produced using the Regional Emission Inventory in Asia for China. Emissions from biomass burning were based on the monthly Global Fire Emissions Database (GFED) version 4 (*73*) for NO_*x*_ and CO. Emissions from soils were based on monthly means of the Global Emissions Inventory Activity (*74*) emissions for NO_*x*_. For other compounds, including VOCs, emissions were taken from the HTAP version 2 and GFED version 4 emissions.

### OPE estimates

The COVID-19 ozone response and OPE were estimated from model simulations using the BAU and 2020 emissions. To estimate the TOB anomaly related to COVID-19, we conducted a model simulation from 1 February 2020 through 31 July 2020 using the initial conditions obtained from a model simulation using the BAU emissions, which provides the accumulated influences of NO_*x*_ emission changes during the course of the COVID pandemic. To evaluate the relative importance of NO_*x*_ emission reductions for each region, additional sensitivity calculations were conducted by separately replacing the BAU emissions with the 2020 emissions for each region (cf. Fig. 7). For estimating OPE (in TgO_3_/TgN), model simulations were conducted from the beginning to the end of each month for February to June 2020, using the same initial conditions at the beginning of each month obtained from a continuous model simulation with the BAU emissions, and the simulated TOB averaged over the last 5 days of each month was compared between the simulations using the BAU and 2020 emissions. This method separately provides monthly changes in the ozone response to reduced NO_*x*_ emissions for each region.

### Statistical analysis

The total uncertainty on the COVID NO_*x*_ emission anomaly includes uncertainties from three sources. The first is the multiyear SD of the BAU emissions. The second is the influence of CTM errors in chemical and physical processes, which was estimated from the multiconstituent chemical data assimilation framework (MOMO-Chem) (*46*) using four forward CTMs in a state-of-the-art ensemble Kalman filter data assimilation system. The third component of the uncertainty is derived from the SDs of the estimated daily emissions within 6 days; this variability is a measure of the uncertainty (stability) of the a posteriori emissions. For most polluted areas, the influence of model errors was comparable to or slightly larger than the BAU multiyear variability, whereas the influence of the short-term variability was much smaller. The total uncertainty is shown in the analysis (Fig. 4). For OPE, the SD of estimated TOB during the analysis period was used to provide an uncertainty estimate. The validation of the model results against assimilated and independent observations is given in the Supplementary Materials (sections S1 to S4).

## SUPPLEMENTARY MATERIALS

Supplementary material for this article is available at http://advances.sciencemag.org/cgi/content/full/7/24/eabf7460/DC1
